# Targeted siRNA Nanoparticles for Mammary Carcinoma Therapy

**DOI:** 10.3390/cancers11040442

**Published:** 2019-03-29

**Authors:** Meital Ben-David-Naim, Arie Dagan, Etty Grad, Gil Aizik, Mirjam M. Nordling-David, Alisa Morss Clyne, Zvi Granot, Gershon Golomb

**Affiliations:** 1 Institute for Drug Research, Faculty of Medicine, The Hebrew University of Jerusalem, Jerusalem 9112001, Israel; meitalben@ekmd.huji.ac.il (M.B.-D.-N.); daganarie@gmail.com (A.D.); ettyg@ekmd.huji.ac.il (E.G.); gil.aizik@gmail.com (G.A.); mirjamd@ekmd.huji.ac.il (M.M.N.-D.); 2 Department of Mechanical Engineering and Mechanics, Drexel University, Philadelphia, PA 19104, USA; alisam@coe.drexel.edu; 3 Institute for Medical Research Israel Canada, Faculty of Medicine, The Hebrew University of Jerusalem, Jerusalem 9112001, Israel; zvikag@ekmd.huji.ac.il

**Keywords:** nanoparticles, targeted delivery system, siRNA, osteopontin, mammary carcinoma

## Abstract

Non-viral, polymeric-based, siRNA nanoparticles (NPs) have been proposed as promising gene delivery systems. Encapsulating siRNA in targeted NPs could confer improved biological stability, extended half-life, enhanced permeability, effective tumor accumulation, and therapy. In this work, a peptide derived from apolipoprotein B100 (ApoB-P), the protein moiety of low-density lipoprotein, was used to target siRNA-loaded PEGylated NPs to the extracellular matrix/proteoglycans (ECM/PGs) of a mammary carcinoma tumor. siRNA against osteopontin (siOPN), a protein involved in breast cancer development and progression, was encapsulated into PEGylated poly(d,l-lactic-co-glycolic acid) (PLGA) NPs using the double emulsion solvent diffusion technique. The NPs obtained possessed desired physicochemical properties including ~200 nm size, a neutral surface charge, and high siOPN loading of ~5 µg/mg. ApoB-P-targeted NPs exhibited both enhanced binding to isolated ECM and internalization by MDA-MB-231 human mammary carcinoma cells, in comparison to non-targeted NPs. Increased accumulation of the targeted NPs was achieved in the primary mammary tumor of mice xenografted with MDA-MB-231 mammary carcinoma cells as well as in the lungs, one of the main sites affected by metastases. siOPN NPs treatment resulted in significant inhibition of tumor growth (similar bioactivity of both formulations), accompanied with significant reduction of OPN mRNA levels (~40% knockdown of mRNA levels). We demonstrated that targeted NPs possessed enhanced tumor accumulation with increased therapeutic potential in mice models of mammary carcinoma.

## 1. Introduction

Polymeric nanoparticles (NPs), formulated with poly(d,l-lactic-co-glycolic acid) (PLGA) copolymer, have emerged as promising carriers for cancer therapy by delivering a wide variety of drugs, including small interfering RNAs (siRNAs) [1,2,3,4,5]. The unique characteristics that make PLGA-based NPs promising candidates for siRNA delivery include their ability to protect the siRNA molecules from degradation, overcoming the cell membrane absorption barrier thereby enabling siRNA internalization into the target cells, tunable sustained release properties, facile possibilities for surface functionalization, and their biocompatibility and biodegradability properties [6,7,8,9]. NPs accumulation in solid tumors is mediated mainly by a passive process (i.e., the enhanced permeability and retention effect (EPR)), based on the leaky vasculature and poor lymphatic drainage present in the tumor [10,11,12,13]. Nanosized particles with neutral surface charges and/or a hydrophilic surfaces (PEGylation) have the propensity for increased circulation time because of decreased phagocytosis by the mononuclear phagocytic system (MPS), which consequently increases their EPR-based tumor accumulation [14]. Their ultimate fate is similar, however, to that of conventional NPs, and the liver/spleen will eventually take up the majority of circulating NPs [15,16]. Hence, for efficient NPs accumulation at the tumor site, a long circulation time and efficient particle targeting are critical. Incorporation of a targeting ligand in NPs’ surfaces enables binding of the carrier with specific targets in the tumor tissue, which most often can be overexpressed, resulting in enhanced tumor accumulation and/or retention [17,18,19]. A broad range of ligands have been used for formulating targeted nanocarriers for enhanced tumor accumulation, including small molecules, carbohydrates, aptamers, peptides, proteins, or antibodies [20].

In our previous studies, PLGA-based NPs, containing antisense [7,21] and siRNAs [22,23] that were neither PEGylated nor targeted, have been examined in mammary carcinoma animal models. In the present study we sought to examine the feasibility of targeting siRNA-containing NPs to the cancer tissue by linking a specific navigator peptide to the NPs surface. In cancer, the tumor vasculature is highly permeable, and the sub-endothelial retention in the extracellular matrix (ECM) of low-density lipoproteins (LDLs) through their interaction with proteoglycans (PGs) is enhanced [24,25,26]. In vitro studies have identified sequences derived from the protein moiety of LDL, apolipoprotein B100 (apoB100), which binds the negatively charged PGs and LDL receptor (LDLr) with a high affinity [27,28,29]. We hypothesized that by linking an apoB100-derived peptide (25 AA; ApoB-P) to the NPs surface [30], the decorated NPs will be targeted to the tumor’s ECM through their interaction with PGs in the tumor’s microenvironment. As a model drug, we used an siRNA sequence specifically designed to knockdown the human osteopontin protein (OPN) [22,23]. OPN, a member of the small integrin-binding ligand N-linked glycoproteins (SIBLINGs) family [31], is considered as a multifunctional protein that plays a central role in malignancy [32,33,34]. Knockdown of OPN expression was shown by us and others to have antimetastatic and antitumorigenic effects [23,35,36,37]. We have shown that siRNA against OPN (siOPN) delivered by NPs inhibits tumor growth in an ectopic model of mammary carcinoma [22]. In this work, we examine whether the use of targeted NPs leads to both enhanced tumor accumulation and superior therapeutic efficacy. We evaluated the targeting efficiency of the ApoB-P NPs in vitro by examining their binding to both isolated basement membrane (BM) and ECM, and their uptake into breast cancer cells. In vivo, we further assessed their biodistribution and bioactivity in mammary carcinoma mice models.

## 2. Results

### 2.1. Poly(d,l-Lactic-Co-Glycolic Acid) Apolipoprotein B100 Peptide (PLGA-ApoB-P) Synthesis

**Figure 1:**
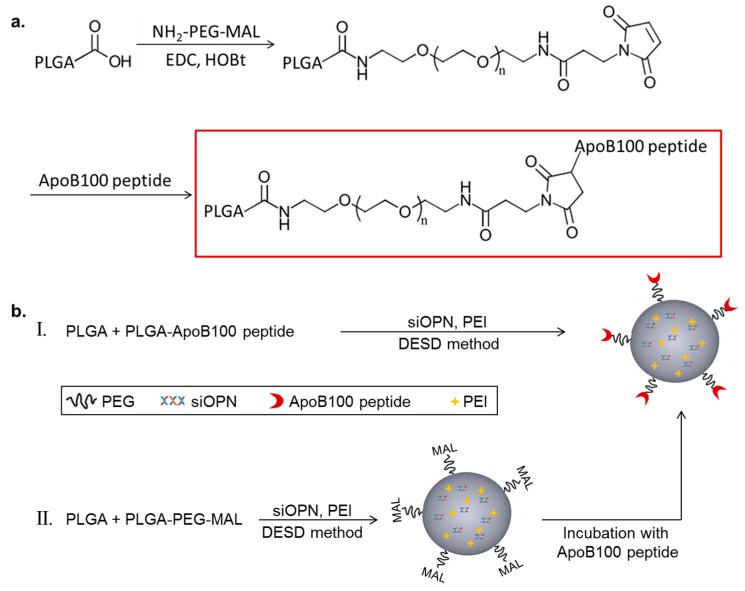
Schematic representation of Polyd,l-lactic-co-glycolic acid) apolipoprotein B100 peptide (PLGA-ApoB-P) synthesis (**a**), and targeted nanoparticle (NP) preparation (**b**), by two methods (I and II).

The targeting peptide, ApoB-P, was linked to PLGA with a polyethylene glycol (PEG) spacer. PLGA-PEG-maleimide (PLGA-PEG-MAL) was synthesized by linking a heterobifunctional PEG containing a maleimide (MAL) group at one terminus to PLGA (Figure 1a). The successful linking between the PEG linker and PLGA was verified by ^1^H NMR spectroscopy (Figure S1). ApoB-P linking in the final step of the synthesis (thiol-maleimide click reaction) was confirmed by amino acid analysis and by elemental analysis. The linking efficiency was >70%.

### 2.2. Nanoparticles (NPs) Physicochemical Properties

Targeted NPs (prepared by either method I or II, Figure 1b) and non-targeted NPs were successfully prepared using the double emulsion solvent diffusion (DESD) method. The NPs had a mean diameter of ~200 nm (empty and siOPN-loaded) with a narrow size distribution and a neutral surface charge (Table 1). siOPN was encapsulated in both targeted and non-targeted NPs, using polyethyleneimine (PEI) of 800 Da as the counter-ion for siOPN complexation, resulting in a relatively high siOPN loading of 4.9 and 5.1 µg/mg, and an encapsulation yield of 29.6% and 31.0%, respectively (Table 1).

### 2.3. In Vitro Binding and Uptake

#### 2.3.1. Binding to Isolated Extracellular Matrix (ECM)

**Figure 2:**
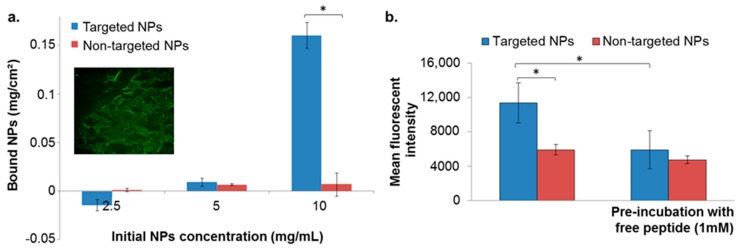
NPs binding to extracellular matrix (ECM) isolated from endothelial cells (**a**), and to basement membrane (**b**). Data is presented as mean ± SD, * *p* < 0.05. Validation of ECM matrix presence in the coated well is shown in the inset (**a**) conducted by fluorescent labeling for heparan sulfate proteoglycan (HSPG) (perlecan protein; basement membrane-specific heparan sulfate proteoglycan core protein).

The binding affinity of targeted NPs to the ECM was evaluated by incubating the NPs with isolated ECM derived from porcine aortic endothelial cells. We validated the presence of ECM in the wells by fluorescent labeling for heparan sulfate proteoglycan (HSPG). No differences between targeted and non-targeted NPs were observed below NPs concentration of 5 mg/mL (Figure 2a). However, at a concentration of 10 mg/mL the targeted NPs showed enhanced binding to the ECM, compared to non-targeted NPs that remained almost undetected (Figure 2a).

#### 2.3.2. Binding to the Basement Membrane (BM) Matrix

The binding affinity of targeted NPs to the BM was evaluated by incubating the NPs in wells pre-coated with Matrigel matrix, using 10 mg/mL of NPs. As in the isolated ECM model, targeted NPs exhibited higher binding affinities in comparison to non-targeted NPs (Figure 2b). Moreover, pre-incubation of the coated wells with free ApoB-P abolished the increased binding of the targeted NPs (Figure 2b). This further supported our hypothesis that the attachment of the NPs was to a specific target in the ECM, which was apparently saturated by the presence of the free ligand.

#### 2.3.3. Uptake into the MDA-MB-231 Cell Line

**Figure 3:**
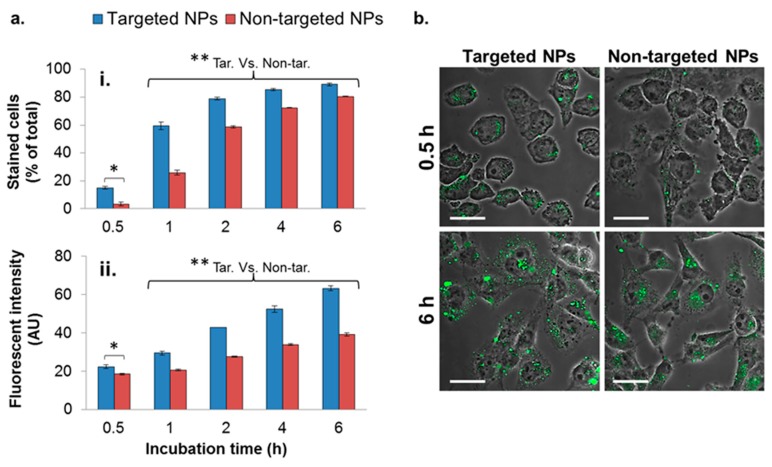
Cellular uptake of NPs by the MDA-MB-231 cell line. Cells internalizing NPs were analyzed quantitatively by means of flow cytometry (FACS) (**a**), and qualitatively by means of confocal laser scanning microscopy (**b**). A total of 10,000 cells were counted in each measurement (*n* = 2). Data is presented as the mean ± SD, * *p* < 0.05 for targeted (Tar) versus non-targeted (Non-tar) at 0.5 h. ** *p* < 0.01 at 1, 2, 4, and 6 h for targeted versus non-targeted comparisons. NPs are shown in green (PLGA-BODIPY); magnification 60×; size bar, 30 µm. The fluorescent intensity was normalized to untreated cells.

To evaluate the enhanced uptake of the targeted NPs into MDA-MB-231 breast cancer cells, targeted and non-targeted fluorescent NPs were incubated with the cells for different durations, and internalized NPs were analyzed by flow cytometry (FACS). The number of cells with internalized targeted NPs was significantly higher at all time points (Figure 3(ai)), in comparison to cells with non-targeted NPs, by a factor of 4.4, 2.3, and 1.3 after 0.5, 1, and 2 h of incubation, respectively. At longer incubation time periods, the difference between the uptakes of targeted and non-targeted NPs declined.

In addition to the higher number of cells internalizing the targeted NPs, each cell engulfed a higher amount of the targeted formulation, demonstrated by the higher fluorescent intensity measured within the cells (Figure 3(aii)). The enhanced uptake of targeted NPs was further observed qualitatively by confocal laser scanning microscopy (Figure 3b), shown by the higher green staining of cells treated with the targeted formulation after 0.5 and 6 h (Figure 3b).

To further examine the mechanism of NPs internalization, targeted and non-targeted NPs were incubated with MDA-MB-231 cells at 4 °C and at 37 °C (Figure S2). Higher uptake of targeted compared to non-targeted NPs was observed at 37 °C (Figure S2). Lowering the temperature significantly reduced both targeted and non-targeted NPs uptakes, suggesting an active and energy-dependent endocytosis (Figure S2). Nevertheless, targeted NPs maintained their higher affinity and uptake at all time points examined, even at reduced temperatures (Figure S2). Of note, after 60 min of incubation, targeted NPs incubated at 4 °C demonstrated higher affinity compared with non-targeted NPs at 37 °C (Figure S2).

### 2.4. In Vivo Biodistribution—4T1 Intravenous (IV) Model

**Figure 4:**
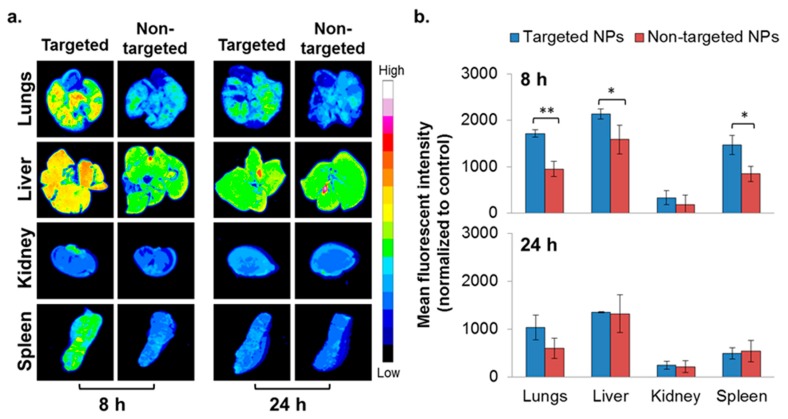
Biodistribution of NPs in the 4T1 intravenous (IV) model. Typhoon images (**a**) followed by ImageJ analysis (**b**) demonstrating targeted (*n* = 3) and non-targeted (*n* = 3) NPs accumulation in different organs. (Non-targeted NPs are described in [22].) The mean fluorescent intensity in each organ was normalized to untreated control. Data is presented as mean ± SD, * *p* < 0.05, and ** *p* < 0.001.

To evaluate the ability of NPs to accumulate at metastatic sites, the 4T1 IV model, a commonly employed model to study breast cancer lung metastasis, was used [38]. Metastatic lesions in the lungs were confirmed using bioluminescence (Figure S3), and the biodistribution of the NPs was studied as reported earlier [22]. Higher accumulation of the targeted NPs in the liver and spleen was noted after 8 h, but was similar after 24 h (Figure 4). There was no difference in the biodistribution of NPs in the kidneys. Significantly higher accumulation of the targeted NPs was observed in the metastatic lungs (1.8 times higher compared to non-targeted NPs) at 8 h after NPs injection (Figure 4). The same trend was observed after 24 h (borderline significance, *p* = 0.07).

### 2.5. In Vivo Biodistribution—Orthotopic Xenograft Model

**Figure 5:**
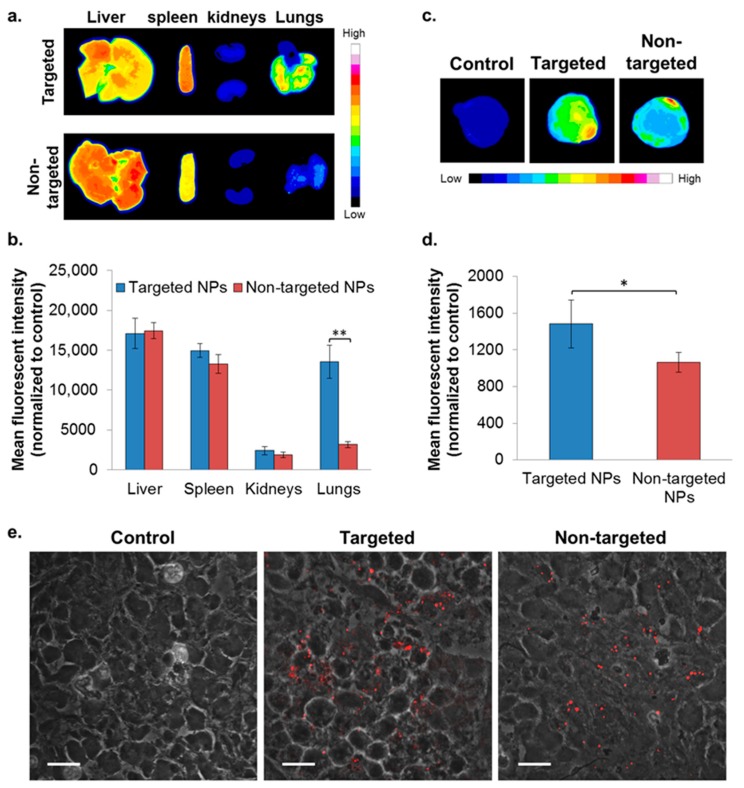
Biodistribution of NPs in the orthotopic xenografted mice model of MDA-MB-231 human mammary carcinoma cells. NPs biodistributions in selected organs (**a**,**b**) and in the primary tumor site (**c**,**d**) following treatment with targeted (*n* = 2) and non-targeted (*n* = 3) NPs was determined by Typhoon images followed by ImageJ analyses, 24 h after IV treatment. The mean fluorescent intensity in each organ was normalized to untreated control (mean ± SD, ** *p* < 0.01, and * *p* < 0.05). Representative confocal microscopy images for qualitative assessment of NPs accumulation in tumor cryosections is shown in (**e**); magnification 60×; size bar, 20 µm. NPs are shown in red (PLGA-Cy5). The fluorescent intensity was normalized to tumor cryosections of untreated control.

To evaluate the capability of the targeted formulation to accumulate in a primary tumor site, biodistribution was studied in the xenograft model of MDA-MB-231 human mammary carcinoma. Targeted NPs accumulated to a greater extent in the primary tumor site, at ~1.4 times higher than the non-targeted NPs (Figure 5c,d). A similar accumulation of targeted and non-targeted NPs was observed in the liver, spleen, and kidneys (Figure 5a,b). Of note, significantly increased uptake of the targeted NPs was observed in the lungs (Figure 5a,b).

### 2.6. In Vivo Bioactivity—Orthotopic Xenograft Model

**Figure 6:**
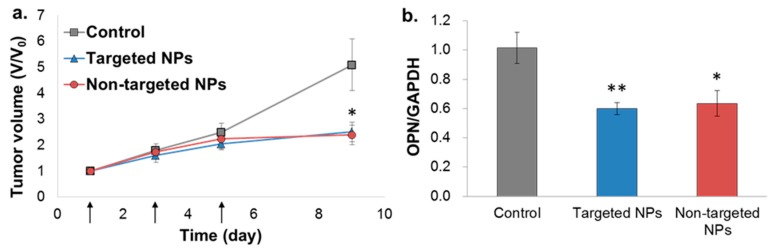
Tumor growth inhibition by siOPN NPs in the orthotopic xenograft MDA-MB-231 mammary carcinoma mice model. Targeted vs. non-targeted siOPN NPs were injected at a dose of 1 mg/kg by body weight of siOPN (arrows, (**a**)). Tumor size is presented as the tumor volume ratio, V/V_0_. V and V_0_, tumor volume measured at each time point and initial tumor volume (day 1), respectively, (mean ± SEM; *n*= 5–7 in each group, * *p* < 0.05, treatment vs. control). Knockdown of OPN mRNA levels evaluated by RT-PCR is shown in (**b**). OPN mRNA levels were normalized to GAPDH and to untreated control animals (mean ± SEM; *n* = 5–7 in each group, * *p* < 0.05, ** *p* < 0.01, treatment vs. control).

The bioactivity of the NPs was evaluated in the orthotopic MDA-MB-231 model, but not in the 4T1 model, because the siOPN sequence used was designed to knockdown human OPN, which is not expressed in the 4T1 cell line (mice origin). Treatment of mice xenografted with MDA-MB-231 human mammary carcinoma cells with targeted and non-targeted siOPN NPs resulted in a similar, significant suppression of tumor growth (Figure 6a). Congruent with significant tumor growth inhibition, a ~40% knockdown of OPN mRNA levels was also observed (Figure 6b).

## 3. Discussion

Polymeric NPs based on the biocompatible and biodegradable PLGA have been explored for the delivery of siRNA [5,22,23,39]. PEGylated NPs, termed “stealth NPs”, are characterized by increased residence times in the circulation and provide increased tumor accumulation via the EPR effect [1,40,41]. Nevertheless, a targeted delivery system (i.e., NPs decorated with a specific tissue/cell ligand) could further increase retention time at the diseased tissue [20,42,43]. In the present study, PLGA-PEG NPs containing siOPN were decorated with a navigator peptide (ApoB-P), which has affinity to PGs in the ECM of the tumor and to LDLr. We demonstrated that targeted NPs possessed significantly enhanced tumor accumulations and increased therapeutic potentials in mice models of mammary carcinoma.

The ligand, ApoB-P, is composed of two consecutive peptide dimers, derived from aa 3145 through 3157 and 3359 through 3367 (of apoB100), linked with a glycine–cysteine–glycine bridge (GCG; 3145–3157-GCG-3359–3367) [30]. The role of the individual dimers in the binding of LDL-apoB100 to PGs and to LDLr has been well established [44,45,46,47]. Olsson et al. [45] have shown that a heterodimer, linked by a glycine tripeptide (3145–3157-GGG-3359–3367), has higher affinity to PGs and LDLr than the two separated segments. We hypothesized that linking the two peptide dimers with the tripeptide, glycine–cysteine–glycine (GCG rather than GGG), could enable facile linking to PLGA-PEG, bestowing a U-shape orientation of the ligand—a conformation resembling that of the native apoB100 protein [27,44,48].

Targeted and non-targeted NPs containing siOPN, prepared by the DESD method, yielded NPs with a size of ~200 nm, a narrow size distribution (low PDI), and a neutral surface charge. The positively charged PEI of 800 Da, which was shown to be less toxic than the routinely used PEI of 25 kDa [22,49], was used as a counter-ion to the negatively charged siRNA in order to achieve efficient encapsulation. Depending on the N:P ratio (cation to anion molar ratio, e.g., the PEI nitrogen to siRNA phosphate ratio), relatively high loading of siOPN was achieved (~5 µg of siOPN/mg of NPs). No difference in the loading of siOPN was found between targeted and non-targeted NPs, suggesting that the presence of PEG-linked ApoB-P to the surface of the NPs did not hamper the encapsulation of siOPN.

The competitive affinity studies, in the two models of BM and isolated ECM (Figure 2), validated the preferred binding of targeted NPs to BM/ECM, indicating their potential to bind and be retained in the tumor’s extracellular space. In addition to the enhanced binding to BM/ECM, the targeted ApoB-P NPs demonstrated an increased uptake into MDA-MB-231 breast cancer cells up to 6 h of incubation (Figure 3). As expected, at longer incubation periods (4 and 6 h), differences between targeted and non-targeted NPs diminished since all the cells eventually engulfed the NPs. The increased uptake of targeted NPs by MDA-MB-231 cells, even at 4 °C (Figure S2), further supports the affinity of the targeted NPs to specific substrate(s) in the cells’ membrane. Cell surface PGs [45] and LDLr, which are upregulated in tumor cells [50,51,52], are the most probable binding sites for the ApoB-P-decorated NPs. Taken together, we postulate that enhanced accumulation of the targeted NPs in the primary tumor could arise from their increased binding/retention at the tumor’s ECM as well as their enhanced uptake into the tumor cells by receptor-mediated endocytosis.

Since Paget’s “seed and soil” hypothesis [53], it is now well established that the lungs are among the primary organs affected in metastatic breast cancer [54,55]. We demonstrate preferred accumulation of the targeted NPs in the metastatic lungs of the 4T1-transplanted mice. A higher accumulation of the targeted NPs in the lungs was also observed in the orthotopic model of MDA-MB-231. Although lung macro-metastases were not observed at the time of sacrifice, micro-metastases are expected to form [56]. Another explanation for the higher accumulation in the lungs of the targeted NPs in the orthotopic model could be due to changes occurring in the pre-metastatic lungs. Evidence has emerged that early influx of neutrophils and other factors (including OPN) secreted by the primary tumor are key mechanisms in establishing the pre-metastatic niche for subsequent engraftment of tumor cells [57], and this leads to increased endothelial permeability and vascular leakiness [58,59]. In addition, PGs, being a major component of the ECM in the alveolar wall [60], are prevalent on the surface of lung capillary endothelium [61]. Nevertheless, the enhanced uptake in the lungs could be, at least in part, due to the abundant interstitial monocytes in this organ [62,63,64]. This is corroborated by the finding (Figure S4) that targeted NPs are engulfed to a higher extent than non-targeted NPs by white blood cells (WBCs), specifically by monocytes. This in turn is most likely because of the different PEGylation type of the NPs. PEG in non-targeted NPs (PLGA-PEG) is expected to be entirely functional in structure in comparison to that in targeted NPs since the PEG moiety is blocked at the end by the peptide (PLGA-PEG-ApoB-P). Taken together, the enhanced accumulation in the lungs of targeted NPs represents a significant potential for lung metastases therapy.

The major MPS organs responsible for particulate system sequestration and disposal are the liver and the spleen [21,65,66]. Indeed, in both mammary carcinoma models, substantial amounts of both targeted and non-targeted NPs were observed in these clearing organs, and similar levels were observed after 24 h (Figure 4 and Figure 5a,b). Similarly, no difference in the disposition of targeted and non-targeted NPs was observed in kidneys, which exhibited the lowest level of biodistribution, probably because the size of the NPs was too large for renal filtration [65].

Finally, a significantly higher amount of the targeted vs. non-targeted NPs (1.4 times) was detected in the primary tumor site (Figure 5c,d). Of note, both targeted and non-targeted NPs were distributed throughout the tumor tissue, located in between the tumor cells (ECM) and inside the cells (Figure 5e). We anticipate that after reducing the uptake of targeted NPs by WBCs, higher levels in the tumor would be obtained. This can be achieved, for example, by adding PEG-PLGA chains in between the ApoB peptide-PEG-PLGA. Nonetheless, their accumulation in the tumor was significantly higher in comparison to non-targeted NPs, indicating their high affinity to the tumor tissue.

There are two possible explanations to the observation that both treatments of targeted and non-targeted siOPN NPs exhibited similar inhibition of tumor growth (Figure 6a). Perhaps the higher levels of targeted NPs detected in the tumor were insufficient to exert a superior therapeutic effect. In addition, retention of the targeted NPs in the ECM of the tumor could have also limited their internalization into the cancer cells, resulting in similar bioactivity as the non-targeted NPs. Nevertheless, it is plausible to assume that the higher levels of targeted NPs found in the tumor could translate to superior efficacy at a later time period (tumor size was measured for a period of nine days since the first NPs injection). The therapeutic effect was in accord with that obtained in an ectopic xenograft model (MDA-MB-231 cells injected SC) treated with non-targeted siOPN NPs [22]. Although siOPN levels in the suppressed tumor were not determined, the therapeutic effect obtained by siOPN NPs was mediated by OPN mRNA knockdown (~40%; Figure 6b), validating our hypothesis. Overall, we developed a new platform for targeted siRNA delivery. This protean platform can be specifically tailored to deliver any siRNA of choice or, for that matter, any other drug intended to inhibit tumor growth. It should be noted that numerous reports describe tumor uptake of drugs following systemic administration by various types of NPs, mainly by the EPR principle [1,10,12,15,16,67,68,69,70]. The fate of nanomedicine has been criticized for lack of effective tumor accumulation [10,62,71,72,73,74,75]. In a recent review summarizing hundreds of studies performed in the field of tumor delivery systems [76], accumulation in the tumor tissue is on average 0.9% and 0.6% of the injected dose (ID), targeted and non-targeted NPs, respectively, and only 0.0014% ID of targeted NPs are detected in the tumor cells [77]. Our study provides a foundation for rationally developing new delivery strategies for cancer therapy.

## 4. Materials and Methods

### 4.1. Materials

PLGA (50:50, ester terminated, MW of 30–60 kDa), tris-EDTA buffer (TE buffer, RNase-free, 10 mM Tris-HCl, 1 mM EDTA), PEI (branched, 800 Da), poly(vinyl alcohol) (PVA, 30–70 kDa), *N*-(3-Dimethylaminopropyl)-N′-ethylcarbodiimide hydrochloride (EDC), 1-Hydroxybenzotriazole (HOBt), and Tris(2-carboxyethyl)phosphine hydrochloride (TCEP) were purchased from Sigma-Aldrich (Rehovot, Israel). PLGA 50:50, acid terminated, MW of 50–60 kDa was purchased from Lakeshore Biomaterials (Birmingham, AL, USA). PLGA-PEG copolymer (RGP d 50105, PLGA, 45 kDa, and PEG, 5 kDa) was purchased from Boehringer Ingelheim (Ingelheim, Germany). The heterobifunctional PEG, amine-PEG-maleimide (NH_2_-PEG-MAL; PEG, 2 kDa) was purchased from Creative PEGWorks (Chapel Hill, NC, USA). siOPN (described in [22,23]) was custom synthesized by Thermo Fisher Scientific (Waltham, MA, USA). The navigator peptide, ApoB-P (a 25 AA sequence: SVKAQWKKNKHRHGCGRLTRKRGLK [30]; MW of ~3 kDa) was purchased from CASLO ApS (Lyngby, Denmark). Mannitol and organic solvents were obtained from J.T. Baker Chemicals (Radnor, PA, USA). Tissue culture reagents and phosphate buffered saline (PBS) were purchased from Biological Industries (Beit-Haemek, Israel).

### 4.2. PLGA-ApoB-P Synthesis

ApoB-P was linked to PLGA through a PEG spacer, as shown in Figure 1a. In the first step of the synthesis, a maleimide-functionalized di-block copolymer, PLGA-PEG-MAL, was synthesized by the conjugation of PLGA-COOH to the bi-functional NH_2_-PEG-MAL. PLGA-COOH (0.017 mmol) dissolved in acetonitrile (ACN) was converted to an active ester using an excess of HOBt (0.7 mmol) and EDC (1 mmol), followed by a reaction with NH_2_-PEG-MAL (0.034 mmol, NH_2_-PEG-MAL/PLGA molar ratio of 2/1). The reaction mixture was left overnight, under constant stirring, at room temperature (RT; 23 °C). PLGA-PEG-MAL was precipitated by the addition of water, followed by centrifugation (4000 rpm, 10 min). PLGA-PEG-MAL was re-dissolved in ACN and precipitated again by the addition of water in order to eliminate residual HOBt, EDC, and unreacted PEG. These washing steps were repeated 3–4 times. After the final washing, the polymer was lyophilized and kept at −20 °C under nitrogen until further use. In order to confirm the linking of PEG-MAL to PLGA, a sample of the resultant polymer was dissolved in deuterated chloroform, and was analyzed by ^1^H NMR (Bruker Avance III 500 MHz NMR). The ^1^H NMR analysis revealed four characteristic peaks, three of them originated from the PLGA at 1.6, 4.8, and 5.2 ppm, corresponding to CH_3_, CH_2_, and CH protons, respectively (Figure S1a,c), and the fourth originated from the PEG protons (CH_2_–CH_2_ protons) at 3.6 ppm (Figure S1a,b). A small peak was observed at 6.7 ppm corresponding to the MAL group protons (Figure S1a,b). These results confirmed the successful linking of the PEG linker as well as the presence of the MAL group, which was essential for further peptide-linking, utilizing a thiol-maleimide click-reaction [78]. In the final step of the synthesis, ApoB-P was reacted with the reducing agent TCEP (10× molar excess of TCEP) in order to break disulfide bonds in the peptide, forming free thiol groups for reacting with the MAL end group. Following 1 h of incubation, the reduced peptide was added (2× molar excess of peptide) to a solution of PLGA-PEG-MAL in ACN/dimethylformamide (DMF) under constant stirring. The reaction was left overnight at RT followed by three washing steps as described above. The final PLGA-ApoB-P was lyophilized and kept at −20 °C until further use. ApoB-P-linking in the final step of the synthesis was confirmed by amino acid analysis (Aminolab Ltd., Nes Ziona, Israel) and by elemental analysis, and the content of nitrogen was derived primarily from the peptide (Analytical Chemistry Lab, The Hebrew University of Jerusalem, Jerusalem, Israel).

### 4.3. Nanoparticles Preparation

The DESD method, previously described by us [22], was employed for preparing siOPN-loaded NPs. A solution of siOPN (1500 µg/mL) in RNase-free TE buffer was emulsified in 3 mL of ethyl acetate (EtAc), containing 90 mg of PLGA and PLGA-PEG-ApoB-P (8:1 weight ratio), and 325 µg PEI, by means of a microtip probe sonicator (Vibra-Cell tip sonicator, Sonic & Materials, Inc., CT, USA), at 20 W output for 90 s. The resulting primary emulsion was further emulsified into a 2% PVA solution (in 10 mL TE buffer), and was sonicated for 90 s at 50% amplitude to form a double emulsion (W/O/W). EtAc was evaporated under reduced pressure using a rotary evaporator (Buchi, Switzerland) resulting in the formation of NPs. NPs were washed twice (TE buffer and double-distilled water) using ultracentrifugation (25,000 rpm, 30 min, 4 °C), re-suspended in a sterile 2% mannitol solution, and lyophilized. Dry lyophilized NPs were stored at −20 °C until use. Fluorescent NPs were prepared by replacing 10% of the PLGA content in the NPs with PLGA-BODIPY (505/515) or PLGA-Cy5, both synthesized in our lab. For comparisons, non-targeted NPs were prepared containing PLGA and PLGA-PEG (8:1 weight ratio as above), and non-pegylated NPs as previously reported [22].

Additional method for preparing ApoB-P targeted NPs was examined by linking the navigator peptide, ApoB-P to pre-formed NPs of PLGA and PLGA-PEG-MAL (Figure 1b, II). The intermediate compound, PLGA-PEG-MAL, and PLGA were dissolved in EtAc, and the NPs were prepared as described above. NPs were washed once (ultracentrifuge) and were then incubated with the free ApoB-P (pre-incubated with TCEP) at a ×2 molar excess. The reaction was kept overnight at 4 °C, NPs were washed (ultracentrifuge) and lyophilized in 2% mannitol.

### 4.4. Determination of NP Size, Polydispersity, and Surface Charge

NPs size, size distribution, and surface charge (ζ potential) were determined by dynamic light scattering at 25 °C (Zetasizer Nano-ZSP, Malvern Instruments, UK) of 1 mg/mL NPs in TE buffer. The size distribution and mean diameter were analyzed by intensity. For each formulation, the mean value was recorded as the average of three measurements.

### 4.5. Determination of siOPN Content

For each batch, accurately weighted ~5 mg of NPs was dissolved in 1.5 mL of 0.5 N NaOH under constant stirring (100 rpm), at 37 °C, until a limpid solution was achieved. Following centrifugation (3000 rpm, 15 min), the supernatant was analyzed by UV spectrophotometry at 260 nm. siOPN concentration was calculated against a suitable calibration curve (degraded siRNA, dissolved in 0.5 N NaOH). Each batch was weighed and assayed in duplicates, and siOPN concentration and encapsulation yield (%) were calculated as previously described [22].

### 4.6. In Vitro Binding Studies

#### 4.6.1. NPs Binding to the Isolated ECM

Porcine aortic endothelial cells were isolated from porcine aortae by the collagenase dispersion method [79], and passages four to nine were used. Cells were maintained in a low glucose Dulbecco’s Modified Eagle’s Medium (DMEM), supplemented with 5% fetal bovine serum (FBS), 1% L-glutamine and 1% penicillin–streptomycin. For ECM isolation, cells were seeded in 96-well plates and cultured for four days. ECM was isolated by 20 mM NH_4_OH containing 0.5% Triton X-100. Solutions of fluorescently-labeled NPs (PLGA-BODIPY; 200 μL, 2.5, 5, and 10 mg/mL; targeted NPs prepared by method I, Figure 1b, I) were added to wells coated with ECM on a rocker, for 2 h at RT. The NPs suspension was aspirated, and the wells were washed three times with PBS. PBS (200 μL) was added to each well, and the fluorescence intensity was measured by means of a microplate reader (ex/em 484/515 nm). The number of NPs that remained bound to the ECM was extracted from a calibration curve.

#### 4.6.2. NPs Binding to the BM Matrix

Plates (96 wells) were coated with a non-gelled protein layer of a Matrigel^®^ matrix (from mouse sarcoma; Corning, Tewksbury, MA, USA), which contained heparan sulfate proteoglycans as a third major component after laminin and collagen IV. Coated wells (thin coating method according to the manufacturer’s protocol) were incubated with either free ApoB-P (1 mM in PBS) or PBS only, for 1 h at 37 °C. The solution of unbound peptide was aspirated, and fluorescently labeled NPs (PLGA-Cy5; targeted NPs prepared by method II, Figure 1b, II) were then added at a concentration of 10 mg/mL and incubated at 37 °C for 4 h. The wells were washed three times with PBS, and 200 μL of PBS were added to each well. Fluorescent intensity was measured using the Typhoon scanner (FLA 9500 biomolecular imager, GE Healthcare, Hatfield, UK) followed by image analysis (ImageJ software, https://imagej.nih.gov).

### 4.7. Cellular Uptake Studies

The human breast adenocarcinoma cell line, MDA-MB-231, was obtained from ATCC (Manassas, VA, USA). Cells were routinely cultivated in RPMI 1640 medium supplemented with 10% FBS, 2 mM L-glutamine, 100 units/mL penicillin, and 100 µg/mL streptomycin at 37 °C and humidified 5% CO_2_ atmosphere.

#### 4.7.1. Quantification of NPs Cellular Uptake

MDA-MB-231 cells were seeded in 12-well plates (0.2 × 10^6^ cells/well) and were left to attach overnight. The following day, the cells were treated with fluorescently labeled NPs (PLGA-BODIPY; targeted NPs prepared by method I, Figure 1b, I) at a concentration of 100 µg/mL and were incubated for 0.5, 1, 2, 4, and 6 h. The cells were washed with PBS three times, harvested, and analyzed for cell-associated NPs by FACS (BDTM LSR II, BD Biosciences, Franklin Lakes, NJ, USA). Non-treated cells were used as controls and were set as a background. The number of stained cells (expressed as the % of total cells) and the fluorescent intensities were calculated based on the obtained FACS histograms (Figure S5), using FCS Express 4 software (De Novo software, Glendale, CA, USA). The energy-dependent uptake was examined by incubating the cells at 37 °C and 4 °C.

#### 4.7.2. Visualization of NPs Cellular Uptake

MDA-MB-231 cells were seeded on coverslips in 12-well plates (0.2 × 10^6^ cells/well) and were left to attach overnight. Cells were incubated for 0.5 and 6 h with 100 µg/mL of fluorescently labeled NPs (PLGA-BODIPY; targeted NPs prepared by method I, Figure 1b, I). The cells were thereafter washed with PBS (×3), fixed using 4% formaldehyde solution for 10 min, washed again with PBS (×3), and mounted onto a microscope slide. Slides were analyzed using an Olympus FV 10i confocal laser scanning microscope (magnification of 60×). Non-treated cells were used as controls and were set as a background.

### 4.8. In Vivo Mice Models

In all in vivo animal experiments, animals were used and treated according to the guidelines of the animal care and use committee of the Hebrew University of Jerusalem (MD-13-13685-5; 1 August 2013 and MD-17-15238-5; 20 October 2017), Israel, and the NIH. The biodistribution of the NPs was evaluated in the 4T1 model of tumor-bearing mice [22] and in the xenograft (human-derived, MDA-MB-231 cells) mammary carcinoma orthotopic mice model. The advantage of the 4T1 model is the formation of lung metastases (Figure S3) as in humans [54]. The bioactivity of siOPN was evaluated in the xenografted MDA-MB-231 mice model since the siOPN sequence, which was designed to human OPN, is inactive in the 4T1 model.

#### 4.8.1. Mammary Carcinoma 4T1 IV Model

4T1 mouse breast cancer cells (ATCC) were cultured in DMEM containing 10% FBS. 4T1 cells, stably expressing firefly luciferase (4T1-Luc) suspended in PBS (1 × 10^5^), were injected intravenously via the tail vein of 6–7 weeks old female BALB/c mice (Envigo Laboratories, Rehovot, Israel). Two weeks after tumor cell injection, metastatic lesions were observed only in the lungs (confirmed by bioluminescence [22], Figure S3). At this time, fluorescently labeled empty NPs (PLGA-BODIPY; targeted NPs prepared by method I, Figure 1b, I) were injected intravenously into the tail vein (10 mg/mice). The mice were sacrificed 8 and 24 h (*n* = 3 in each group) post-injection, perfused with PBS, and the lungs, liver, kidney, and spleen were harvested. Accumulation of the fluorescent NPs in the organs was evaluated by fluorescent imaging (Typhoon FLA 9500 biomolecular imager, GE Healthcare) followed by image analysis (ImageJ software). The mean fluorescent intensity in each organ was normalized to an untreated control (organ autofluorescence). To evaluate the fate of NPs in the circulation, 8 h after NPs injection, heparinized blood was drawn by cardiac puncture under anesthesia. The red blood cells were lysed (Erythrolyse, AbD, Serotec, Oxford, UK), and the pellet was washed twice with FACS buffer (1% BSA in PBS). Samples were analyzed for cell-associated NPs by FACS (BDTM LSR II), and FCS Express 4 software was used for quantitative analysis. The different populations of WBCs were gated according to their typical forward (size) and side (granularity) scattering, and the fluorescence of the gated cells was measured. The percentage of positive fluorescent WBCs and monocytes was extracted. Data were presented as mean ± SD.

#### 4.8.2. Mammary Carcinoma Orthotopic Mice Model

The biodistribution and bioactivity of targeted and non-targeted NPs were evaluated in the orthotopic model of mice xenografted with MDA-MB-231 mammary carcinoma cells [80]. Mammary carcinoma cells (1 × 10^6^ in 50 µL PBS) were injected into the inguinal mammary fat pad of 6–7 weeks old female athymic nude mice (Envigo). The tumor was visible 2–3 weeks after transplantation, measured externally by a caliper. Mice with similar tumor sizes were randomly assigned to treatments of targeted NPs vs. non-targeted NPs.

Empty NPs, fluorescently labeled with Cy5, were used for evaluating the accumulation of NPs in the tumor and selected organs (lungs, liver, kidneys, and spleen) and their uptake by WBC. Targeted (*n* = 2; NPs prepared by method II, Figure 1b, II) and non-targeted (*n* = 3) NPs (10 mg/mice) were intravenously injected via the tail vein. The mice were sacrificed 24 h post-injection, and the NPs fluorescent signal in the harvested organs was determined by means of fluorescent imaging (Typhoon FLA 9500 biomolecular imager) followed by image analysis (ImageJ software). The mean fluorescent intensity in each organ was normalized to an untreated control (organ autofluorescence). In addition, tumor cryo-sections were visualized by confocal laser scanning microscopy. Immediately after Typhoon scanning, the tumors were embedded in OCT (Bar-Naor, Ramat Gan, Israel) followed by snap-freezing in liquid nitrogen, and stored at −80 °C for further cryo-sectioning (CM1950 cryostat, Leica Biosystems, Wetzlar, Germany). Tumor cryo-sections were performed at a 10 µm width, washed with PBS, fixed (4% formaldehyde), mounted, and visualized using an Olympus FV10I confocal laser scanning microscope. The fluorescent intensity was normalized to cryo-sections of untreated mice. For evaluating the NPs uptake by WBC, blood was collected (24 h post-injection) and analyzed for cell-associated NPs by FACS, as described above.

The bioactivity of siOPN NPs was evaluated in the mice orthotopic xenograft model since the siOPN sequence was designed for human OPN (expressed in the human-originated MDA-MB-231 cells, but not in the 4T1 cells, which are of mice origin). MDA-MB-231 cells were transplanted and examined as mentioned above. Mice having similar tumor sizes were randomly assigned to the different treatment groups and treated with either targeted siOPN NPs (*n* = 7; NPs prepared by method II, Figure 1b, II), non-targeted siOPN NPs (*n* = 5), or left untreated (*n* = 5). The NPs were intravenously injected (tail vein) at a siOPN dose of 1 mg/kg of body weight, every other day for a total of three injections. The tumor size was measured, blinded to the operator, externally by a caliper for a period of nine days after the first injection. Tumor volume was calculated by the formula ½ × *L* × *W*^2^, where *L* is the length (in cm) and *W* is the width (cm).

For determining the mechanism of siOPN treatment, OPN mRNA levels in the excised tumors were analyzed using real-time PCR (RT-PCR). Tumor samples were embedded in 1 mL TRI reagent (Sigma-Aldrich), homogenized, and the total RNA was isolated according to the manufacturer’s protocol. For each sample, RNA concentration was determined by means of a NanoDrop 1000 spectrophotometer (Thermo Fisher Scientific). cDNA was synthesized from 1 µg of extracted RNA using the Moloney murine leukemia virus reverse transcriptase (M-MLV RT, Promega, Madison, WI, USA), and oligo dT primer (Promega). mRNA levels of OPN and GAPDH (housekeeping gene) were quantified by SYBR green-based quantitative RT-PCR (qRT-PCR), performed using the CFX Connect™ Real-Time PCR Detection System (Bio-Rad, Hercules, CA, USA). The following primers were used: human OPN, forward: 5′-CGC AGA CCT GAC ATC CAG T-3′, reverse: 5′-GGC TGT CCC AAT CAG AAG G-3′; human GAPDH, forward: 5′-TCA AGC TCA TTT CCT GGT ATG-3′, reverse: 5′-GTG GTC CAG GGG TCT TAC TC-3′. Thermal cycling parameters for amplification were: 95 °C for 10 min, followed by 40 cycles of 95 °C for 5 s, and 60 °C for 15 s. OPN mRNA levels were normalized to GAPDH and to untreated control mice. Each cDNA sample was measured in triplicate, and mean cycle threshold (Ct) values were reported. ΔCt of each sample was calculated as follows: Ct of the target gene (OPN) minus Ct of the reference gene (GAPDH, housekeeping gene). Then, the mean ΔCt of the untreated control mice was chosen as the reference for the relative quantification calculation (2^−^^ΔΔ^^Ct^). Data were expressed as OPN/GAPDH (mean ±  SEM). 

### 4.9. Statistical Analysis

Data was expressed as the mean ± standard deviation/error. For statistical analysis, the Student’s *t*-test for independent means was used. Differences were termed significant at *p* < 0.05.

## 5. Conclusions

PLGA-PEG NPs containing siOPN, decorated with ApoB-P as a targeting ligand to the tumor, were successfully formulated. Because of the high affinity to ApoB substrates, both increased ECM binding and cellular uptake were obtained. Biodistribution studies revealed enhanced accumulation in the metastatic lungs of mice mammary carcinoma models (4T1 transplantable breast tumor, and orthotopic MDA-MB-231 mammary carcinoma). Despite the significantly higher retention of siOPN NPs in the tumor following intravenous treatment with targeted NPs, a similar therapeutic effect resulted following treatment with non-targeted siOPN NPs. It is suggested that further improvement of the targeting could be of value, and/or that a longer observation time is required. The obtained significant tumor growth suppression was accompanied by a significant reduction of OPN mRNA levels. This validates our hypothesis that systemically administered ApoB-P-targeted siOPN NPs could inhibit tumor progression by inhibiting OPN.

## Figures and Tables

**Table 1:** Physicochemical properties of targeted and non-targeted NPs (mean ± SD).

NP Type	Size (d., nm)	Polydispersity Index (PDI)	Surface Charge (ζ Potential, mV)	siOPN Loading (µg/mg NPs)	Encapsulation Yield (%)
Targeted empty	212.4 ± 3.6	0.21 ± 0.02	−0.9 ± 0.2	-	-
Non-targeted empty	207.3 ± 4.3	0.17 ± 0.02	−1.1 ± 0.1	-	-
Targeted siOPN	233.9 ± 3.7	0.29 ± 0.01	−0.6 ± 0.2	4.9 ± 0.2	29.6 ± 0.9
Non-targeted siOPN	228.5 ± 23.0	0.31 ± 0.04	−0.5 ± 0.1	5.1 ± 0.2	31.0 ± 1.4

## Supplementary Materials

The following are available online at https://www.mdpi.com/2072-6694/11/4/442/s1, Figure S1: Confirmation of amine-PEG-maleimide (NH2-PEG-MAL) conjugation to PLGA by 1H-NMR analysis, Figure S2: Temperature-dependent uptake of targeted and non-targeted NPs by MDA-MB-231 cell line, Figure S3: A representative image of the metastatic lungs in the 4T1 IV model, Figure S4: NPs uptake by circulating WBC and monocytes, examined in the 4T1 IV model and in the orthotopic xenograft model, Figure S5: Cellular uptake of NPs by MDA-MB-231 cell line.
