# Supplementary material for: Targeted siRNA Nanoparticles for Mammary Carcinoma Therapy
Source: Cancers (Basel). 2019 Mar 29;11(4):442. doi: 10.3390/cancers11040442 (PMC6521050; doi:10.3390/cancers11040442)
Supplement: Supplementary file 1 [file cancers-11-00442-s001.pdf]

Supplementary Materials: Targeted siRNA Nanoparticles for Mammary Carcinoma Therapy

Meital Ben-David-Naim, Arie Dagan, Etty Grad, Gil Aizik, Mirjam M. Nordling-David, Alisa Morss Clyne, Zvi Granot and Gershon Golomb

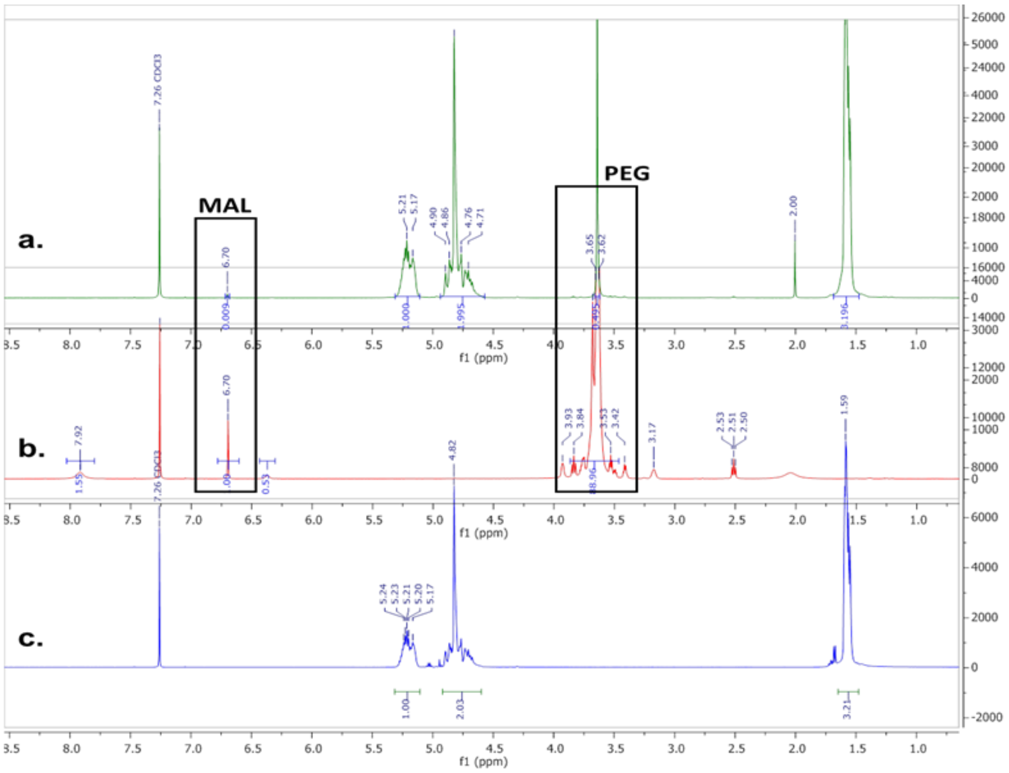

Figure S1. Confirmation of amine-PEG-maleimide (NH_2-PEG-MAL) conjugation to PLGA by ^{1}H-NMR analysis. PLGA-PEG-MAL (**a**); NH_2-PEG-MAL linker (**b**); PLGA (**c**).

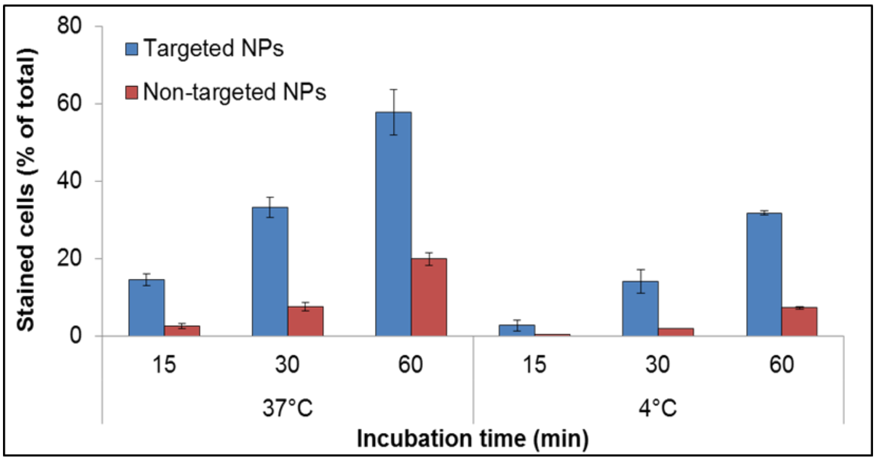

Figure S2. Temperature-dependent uptake of targeted and non-targeted NPs by MDA-MB-231 cell line. Cells were analyzed for internalized NPs by means of FACS. The fluorescent intensity was normalized to untreated cells. A total of 10,000 cells were counted in each measurement (n = 2). Data is presented as the mean ± SD; ** $p < 0.01$ for all time-points (except for 15 min at 4 °C). The effect of lowering temperature on cellular uptake was more pronounced on non-targeted NPs uptake (6.2-, 3.8- and 2.7-fold, and 5.2-, 2.3- and 1.8-fold, after 15, 30 and 60 min, non-targeted NPs and targeted NPs, respectively).

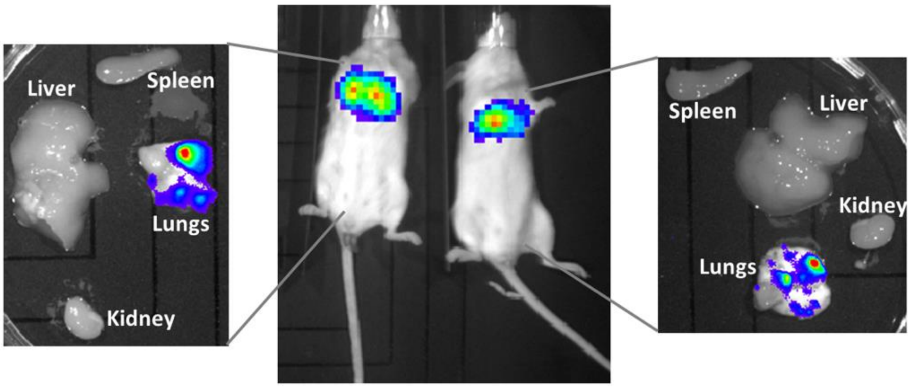

Figure S3. A representative image of the metastatic lungs in the 4T1 IV model. Two weeks after tail-vein injection of tumor cells, mice were injected intraperitoneally with D-luciferin and were imaged by an IVIS machine to confirm metastases formation. Metastases were formed exclusively in the lungs.

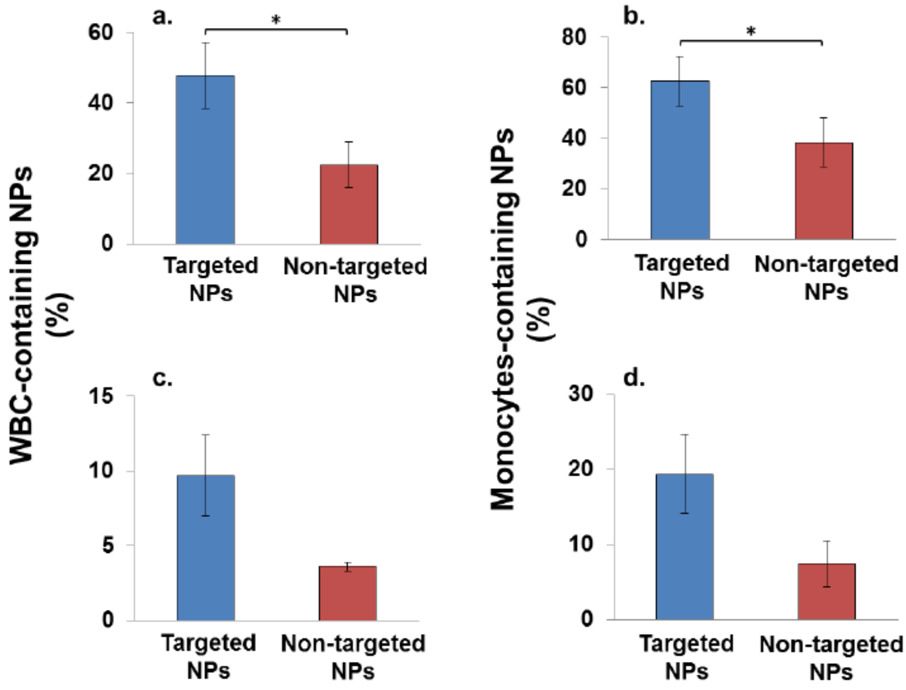

Figure S4. NPs uptake by circulating WBC and monocytes, examined in the 4T1 IV model (8 h after NPs injection; **a**,**b**) and in the mammary fat pad MDA-MD-231 xenograft model (24 h after NPs injection; **c**,**d**). Blood was collected and analyzed by means of FACS for cell associated NPs. The number of positively-stained cells for NPs is shown as percent of WBC and of monocytes- containing NPs. Data is presented as mean ± SD (n = 4 and n = 2, 4T1 IV model and MDA-MD-231 xenograft model, respectively; * $p < 0.05$).

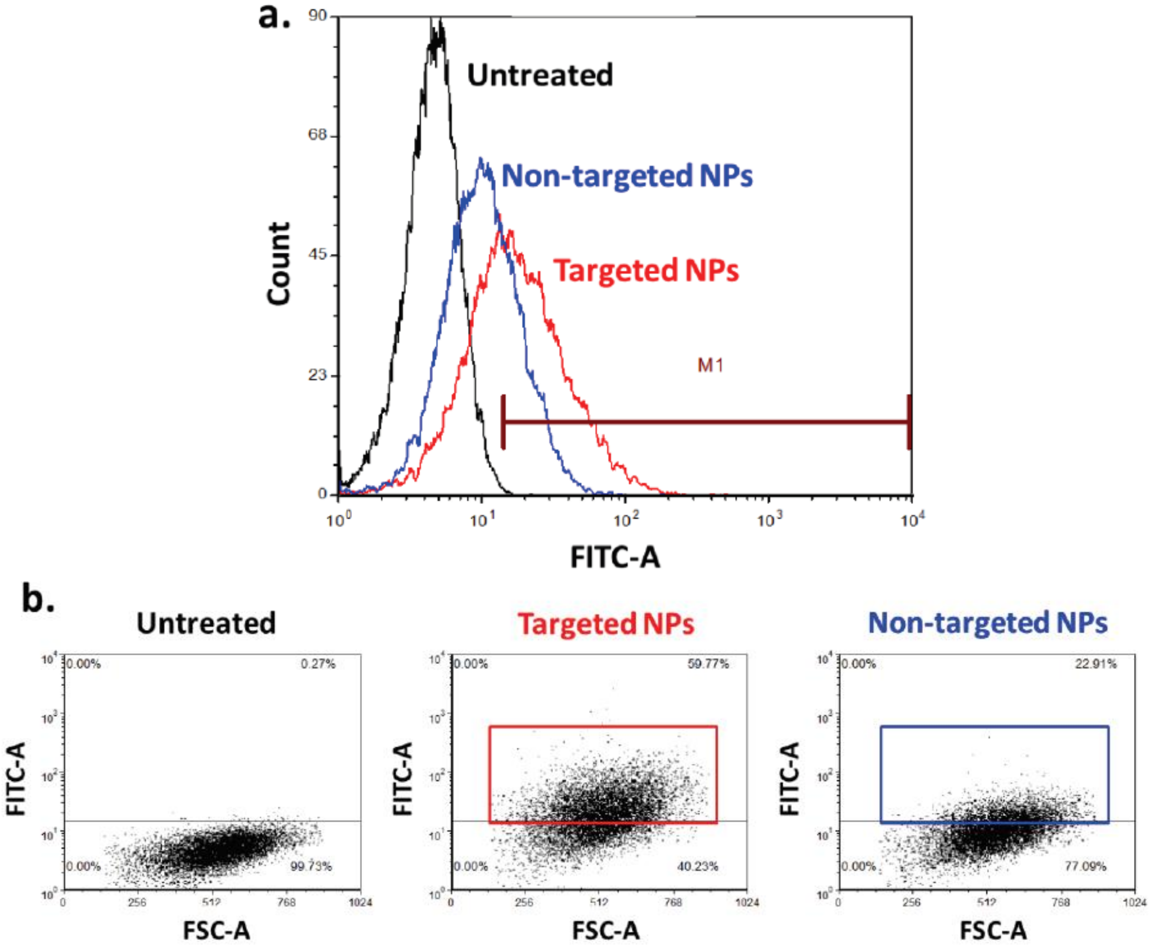

Figure S5. Cellular uptake of NPs by MDA-MB-231 cell line. Representative histograms (**a**) and dot-plots (**b**) obtained using FACS analyses. The data presented is of NPs' uptake following 1 h of incubation with the tumor cells.

© 2019 by the authors. Licensee MDPI, Basel, Switzerland. This article is an open access article distributed under the terms and conditions of the Creative Commons Attribution (CC BY) license (http://creativecommons.org/licenses/by/4.0/).
